# Experiences of family caregivers taking care getting lost of persons with dementia: a qualitative study

**DOI:** 10.1186/s12888-024-05891-0

**Published:** 2024-06-18

**Authors:** Shu-Hui Li, Shu-Fang Vivienne Wu, Chieh-Yu Liu, Chiou-Fen Lin, Hung-Ru Lin

**Affiliations:** 1 School of Nursing, National Taipei University of Nursing and Health Sciences, No. 365, MingTe Rd, PeiTou, District, Taipei, 112303 Taiwan; 2 Department of Nursing, Hsin Sheng Junior College of Medical Care and Management, Taoyuan, Taiwan; 3 Department of Health Care Management, National Taipei University of Nursing and Health Sciences, Taipei, Taiwan; 4 School of Gerontology and Long-Term Care, College of Nursing, Taipei Medical University, Taipei, Taiwan; 5 Department of Nursing, Shuang Ho Hospital, Taipei Medical University, Taipei, Taiwan

**Keywords:** Dementia, Getting lost, Family caregivers, Experiences of taking care, Qualitative study

## Abstract

**Background:**

Getting lost with family members who have dementia is a significant source of stress for family caregivers. In Taiwan, family caregivers develop strategies to deal with dementia persons who may get lost. This study aimed to explore the experiences of family caregivers caring for persons with dementia who have been lost outside the home.

**Methods:**

A descriptive phenomenological method was used. The COREQ checklist was used to ensure the explicit reporting of data. A total of 20 family caregivers caring for persons with dementia who were lost outside their homes were selected from hospital outpatient clinics and a day care center in northern Taiwan using purposive sampling. Data were analyzed using the Giorgi analysis method.

**Results:**

Five main themes emerged: (i) surprised persons with dementia lost outside, (ii) using strategies to prevent persons with dementia from getting lost, (iii) using strategies to find lost persons with dementia, (iv) exhaustion in long-term care persons with dementia, and (v) coping with the care load. It was found that family caregivers were surprised, nervous, and worried about persons with dementia being lost outside. They used the first strategy to supervise persons with dementia to prevent external losses. In addition, long-term supervision of persons with dementia led to mental exhaustion in the family caregivers. Finally, the family caregivers learned about loss prevention strategies and obtained family support and care replacement workers to reduce the care burden.

**Conclusions:**

It is essential to teach family caregivers early to prevent persons with dementia from losing external strategies. Nurses also provide long-term care services to reduce the care burden on family caregivers.

## Introduction

According to World Health Organization (WHO) statistics, dementia ranks seventh among the top ten causes of death worldwide. More than 55 million people worldwide have dementia, with approximately 10 million new cases diagnosed every year [1]. With an increasing international population with dementia, Taiwan faces the same dilemma. In 2019, Taiwan had 235,000 people seeking medical treatment for dementia, an annual increase of 15,000 [2].

Dementia is characterized by impaired memory function and cognitive abnormalities accompanied by increased attention to behavioral disturbances [3]. Getting lost is one of the early symptoms of dementia, with most people experiencing it for the first time within two years of diagnosis [4, 5]. Researchers propose that loss presents navigational deficits related to landmark recognition or executive functioning [6, 7]. Being lost means that the individual involved cannot find either their way, implying a state of spatial disorientation that can occur inside or outside the boundaries of the care setting and either in proximity to a residential setting or outside the community [8]. Loss often occurs when persons with dementia perform normal and permissible activities alone, and the occurrence of loss is unpredictable [9]. By understanding the common situations and the signs that lead to getting lost, family caregivers can be reminded to be more attentive. Loss occasionally occurs repeatedly. In Taiwan, the incidence rate of getting lost in communities is 33.3%, and the recurrence rate of getting lost is 40% [10]. Losing persons with dementia from home is potentially dangerous and increases the risk of falls, physical injuries, and death [11].

Previous studies have shown that issues related to caring for a lost family member include many dimensions, such as the situation of getting lost, family caregivers’ emotional and behavioral reactions to facing a lost family member, and the impact on lives caused by caring for a lost family member. Obtained loss events can occur at any stage of dementia, and family caregivers are unprepared for the sudden occurrence of a loss event [12]. After persons with dementia suffer from the first loss event, it brings excellent emotional stress and fright to family caregivers [13]. Obtaining losses is one of the most critical and challenging management aspects of persons with dementia, increasing the burden on family caregivers [14]. Therefore, loss may have additional consequences for persons with dementia, such as loss of independence. Family caregivers lock persons with dementia inside and restrict their movement outside [14], and they are always accompanied by someone when they go out [10]. However, some family caregivers use global positioning systems (GPS) to track persons with dementia [15]. In contrast, although a quarter of persons with dementia had lost their way, the family caregivers did not realize the risk of getting lost in persons with dementia, and only half of the family caregivers believed that persons with dementia might be lost because of severe memory problems. However, few family caregivers have taken measures to prevent potentially lost events [16]. Despite technological advancements and the continuous development of strategies to prevent persons with dementia from failing, the risk of losing has not entirely disappeared. Persons with dementia still have repeated incidents of getting lost [10]. It is worth exploring how Taiwanese family caregivers react to and deal with the situation when persons with dementia get lost.

Under the traditional concept of filial piety, family members play the role of long-term care providers and take on care responsibilities. Care issues can be prevented by promoting the “discovery and utilization of resources,” which helps caregivers maintain stable daily routines and continue providing care [17]. Formal or informal help from others and access to dementia care and healthcare service-related information are important needs of family caregivers [18, 19]. When family caregivers do not have the support of others, they lack time for self-care, which can result in a perceived burden, an increased likelihood of depression, and a decreased quality of life [18]. Therefore, understanding and meeting the needs of family caregivers is crucial for reducing their caregiving burden.

Neubauer and Liu [20] developed a conceptual model on strategy adoption for dementia and wayfinding. The model provides a series of guidelines to assist persons with dementia and family caregivers who are at risk of getting lost in adopting high-tech and low-tech wandering management strategies. Although the guidelines provide a high-quality review of the evidence, the recommendations lack behavioral specificity and are lengthy, making it challenging for family caregivers to locate relevant information. However, are the developed guidelines directly applicable to Taiwan? Different social cultures, family cultures, and values may lead to varying attitudes. Second, Taiwan currently lacks strategies to assist family caregivers in managing the getting lost behaviors of persons with dementia [21]. Therefore, there is necessary to understand the experiences of family caregivers in caring for lost persons with dementia from the perspective of family caregivers and find strategies suitable for families to deal with persons with dementia. When designing strategies to prevent loss or develop related policies, it is necessary to include family caregivers’ experience in caring for lost persons so that strategies for preventing loss and policies can better meet the needs of dementia caregivers.

This study explored the experiences of family caregivers caring for persons with dementia who were lost outside their homes.

## Methods

This study uses a descriptive phenomenological research method. Descriptive phenomenology stimulates the perception of lived experiences while emphasizing the richness, breadth, and depth of these experiences, it pertains the three-step process for descriptive phenomenology: (1) intuiting, the researcher avoids all criticism, evaluation, or opinion and requires total immersion in the phenomenon of family caregivers’ experiences in the context of persons with dementia getting lost; (2) analyzing, phenomenological analysis involves identifying the essence of the phenomenon under investigation based on the data obtained and how the data are presented; and (3) describing, in the descriptive stage, researchers focus on understanding and defining the phenomenon of family caregivers’ experiences of persons with dementia getting lost. This aims to provide the final step that enhances communication by offering clear distinctions and critical descriptions in both written and verbal forms [22]. Semi-structured interviews were used to guide the in-depth interviews. The design of the interview guide (Table 1) was based on the researchers’ accumulated experiences in participating in Community-Based Dementia Service Stations, understanding of dementia, reading relevant literature, and seeking qualitative expert guidance. Understanding the experiences of family caregivers caring for persons with dementia who were lost outside their homes and the nature of family caregivers’ care experiences was essential.

**Table 1:** Interview guidelines

1.	Please tell me about your situation when your family member is getting lost.
2.	What was your reaction?
3.	How did you deal with it?
4.	What were the signs that family members had before they were lost?
5.	What troubles did you encounter in caring for a lost family member?
6.	How has your life changed since caring for a lost family member?
7.	What kind of assistance did you hope to get?
8.	Do you have any other experiences that you can share with me?

### Participants and setting

For the purposive sampling method used in this study, family caregivers of persons with dementia were recruited from outpatient departments of a 1000-bed regional teaching hospital and a community day care center caring for 44 elderly people with dementia in northern Taiwan. The inclusion criteria for the family caregivers were as follows: (1) the person being cared for by the family caregiver, who has a Clinical Dementia Rating (CDR) of 1 or above, has been diagnosed with dementia by a medical doctor and had experienced getting lost (a situation in which the person could not find his intended destination, way home, or point of departure). Persons with dementia without walking impairment, brain injury, or visual impairment; (2) responsible for taking care of persons with dementia; (3) currently participating in care activities or care arrangements for family members with persons with dementia for more than 3 months; (4) living with persons with dementia; and (5) communicating in Mandarin or Taiwanese and expressing their opinions concretely. Exclusion criteria include: (1) the person being cared for by the family caregiver, who only having wandering locomotion behavior which characterized by repetitive activity, but never appear the getting lost behavior the inability to find one’s way in familiar or unfamiliar environments, and (2) family caregivers with physical and mental health limitations that impede their ability to comprehend the assessment content or respond accurately, such as family caregivers with mental disorders, autism, dementia, intellectual disabilities, etc.

### Ethical considerations

This study was reviewed and approved by the Institutional Review Board of the Northern Hospitals in Taiwan (No. 202200584BO, 111-D-05-01). Participants were interviewed in an empty outpatient clinic in a quiet environment, which also ensured their privacy. Pseudonyms were used during the transcription of the interviews to protect the identity of each participant, ensuring that third parties could not link them to the study. Before conducting the research, the researcher explained the study’s purpose, process, and methods and asked participants to sign a consent form. The participants had the right to withdraw from the study at any time and under any circumstance.

### Data collection procedure

The data collection period for this study is from July 2022 to February 2023. Interviews were conducted in an empty outpatient clinic, providing a private and undisturbed space. The researcher explained the purpose of the study and invited participants to take part. Interviews were conducted by one researcher using semi-structured interview guidelines to conduct personal in-depth interviews. All participants were audio-recorded, and the data was supplemented with field notes. Depending on the richness of the interview content, each participant received a 45-60-minute audio-recorded interview. Each participant was interviewed once. After completing all the questions, they also asked, “Do you have any other experiences that you can share with me?” The researcher summarized the content of the interview and confirmed it with the participant. By interviewing various participants, the care experience of family caregivers of persons with dementia who exhibit getting lost behavior can be more comprehensive, and the data were considered saturated and sufficient when participants’ lived experiences began to repeat, and no new themes emerged.

### Data analysis

The transcribed interviews were analyzed according to Giorgi’s phenomenological analysis strategy [23]. This analysis consists of four steps: (1) the researcher read the text several times, feeling and understanding participants’ experiences to obtain a general sense of the whole; (2) the researcher went back and reread the text, and the text was divided into smaller units, meaning units, with a focus on the phenomenon being researched; (3) for all meaning units, the researcher integrates the main points or similar attributes that appear repeatedly in several meaning units into themes; and (4) obtain the interrelatedness between themes, which constitutes a theme synthesis as a complete description of the experienced situated structure.

### Trustworthiness

The rigor of this study was based on the four standards of trustworthiness of Lincoln and Guba’s [24] credibility, dependability, confirmability, and transferability to evaluate the accuracy of the data. To ensure credibility, persons with dementia and their families often endure long waits in the outpatient waiting area before consulting with doctors. Researchers often communicate with them in the waiting area and maintain long-term contact with them. This approach can help establish a trusting relationship with persons with dementia and their families. The entire interview process was audio-recorded, and non-verbal cues and situational messages during the interview were also recorded, enabling participants to express their thoughts freely. After the interview, the researcher verified the interview content with the participants. Afterwards, the results are further analyzed and discussed with the professor to minimize errors in the analysis. For dependability, the data collection was conducted by a researcher. After interviewing each family caregiver, the verbatim manuscripts were analyzed and discussed with the professor. Notes were taken at any time to identify areas where interviewing skills needed improvement, with the aim of enhancing them for future interviews. To enhance sensitivity to data. To ensure confirmability, the researcher prioritized listening over speaking during interviews. This strategy aimed to create a non-judgmental environment that encouraged participants to freely express themselves. Additionally, the researcher employed empathy skills to accurately convey the perspectives of the participants. During the data analysis process, researchers used reflection journals to reflect on their potential biases. They made efforts to adjust and control these biases to ensure that the research results were impartial and aligned with objectivity. Regarding transferability, the richness of the data increased. Purposive sampling was adopted, and the participants included males and females, including spouses and children, as the family caregivers, regardless of age, to collect the rich care experience of caregivers of all ages. The entire interview was recorded with a tape recorder to reduce the data’s mission and increase the data’s richness.

## Results

A total of 20 family caregivers in this study took care of persons with dementia who had experienced getting lost, with an average age of 60.4 years (22–87 years old); 15 were females, 55% of the family caregivers were children, 35% had a job, 50% took care of persons with dementia for more than 17 h a day, and 40% felt that their health had worsened. The average age of the persons with dementia was 79.2 years old, 45% had a Clinical Dementia Rating (CDR) of 2, and the average duration of illness was 5.15 years (Table 2).

The results of this study present the essential structure of dementia family caregivers’ experiences of caring for lost family members. Five themes emerged:

**Table 2:** Basic information of family caregivers and persons with dementia

Family caregivers								Persons with dementia			
ID	Sex	Age	Relation	Daily care time (hours)	Health status	Employment status		Sex	Age	CDR	Disease time (year)
P1	Female	51	Daughter	4–8	Unaffected	Full time		Female	75	2	10
P2	Female	56	Daughter-in-law	>17	Worsened	Unemployed		Female	83	3	10
P3	Male	59	Son	4–8	Unaffected	Full time		Female	83	2	7
P4	Female	22	Granddaughter	>17	Unaffected	Unemployed		Female	71	3	2
P5	Female	64	Daughter-in-law	>17	Unaffected	Unemployed		Female	92	3	2
P6	Male	73	Husband	4–8	Unaffected	Unemployed		Female	71	2	3
P7	Male	45	Son	<4	Unaffected	Full time		Female	93	1	3
P8	Female	68	Daughter-in-law	>17	Unaffected	Unemployed		Female	99	3	2
P9	Female	64	Wife	4–8	Unaffected	Unemployed		Male	74	1	5
P10	Female	51	Daughter	4–8	Worsened	Unemployed		Male	73	2	4
P11	Female	43	Daughter	<4	Unaffected	Full time		Female	71	1	5
P12	Female	51	Wife	<4	Worsened	Full time		Male	55	2	4
P13	Female	62	Daughter	>17	Worsened	Unemployed		Female	84	1	2
P14	Male	73	Husband	>17	Unaffected	Unemployed		Female	72	2	10
P15	Female	46	Daughter	13–16	Worsened	Full time		Female	80	3	4
P16	Female	83	Wife	>17	Worsened	Unemployed		Male	88	1	3
P17	Male	87	Husband	>17	Unaffected	Unemployed		Female	85	2	10
P18	Female	76	Wife	>17	Worsened	Unemployed		Male	87	2	5
P19	Female	72	Wife	>17	Unaffected	Unemployed		Male	74	1	4
P20	Female	62	Daughter	4–8	Worsened	Full time		Male	82	2	8

### Theme 1. Surprised persons with dementia lost outside

The family caregivers found that the persons with dementia had disappeared at home or had not returned to familiar places. As they looked for the lost person, they experienced an emotional change. This theme includes three subthemes.

#### Surprised persons with dementia disappear unexpectedly

Persons with dementia are lost unexpectedly, usually in places they frequently visit daily, in outdoor toilets, or when they leave at night and lose their way. Even when accompanied, attentive persons can suddenly disappear, alarming the family caregivers.

“Once, my mother went out with my father. My father was going to do some shopping and asked my mother to wait, but my mother promised to wait for him, but she left immediately, ……My father just stood there, not knowing what to do.” (P6).

#### Nervously looking for persons with dementia

Family caregivers were very nervous and worried when they were looking for lost persons with dementia.

“My mother-in-law was lost. We spent 2 h searching in the TAIPEI EXPO PARK. We searched all the toilets and exhibition halls. However, we did not find any such cases. I was very nervous…. I did not know where she went.” (P11).

#### Worried about persons with dementia getting lost or having accidents

While looking for lost persons with dementia, the family members worried that the person would have a car accident or fall.

”I was worried that he would get into a car accident when he went out and lost his way. I was terrified. Every time he went on an expressway, it was unexpected for us. We did not know he was on an expressway until the highway police informed us.” (P14).

### Theme 2. Using strategies to prevent persons with dementia from getting lost

For the persons with dementia who were lost at any time, the family caregivers used strategies to prevent loss according to family habits. This theme contains three subthemes.

#### Someone supervises the persons with dementia at all times

After the persons with dementia were lost, the family caregivers always accompanied the persons to prevent them from becoming lost again.

“I realized that she did not know the home way and was worried that she would disappear. Thus, I did not dare to let her go out alone. She had to have family members accompany her. I thought this was the most fundamental and best way to go out with her.” (P1).

#### Set up confinement to prevent persons with dementia from going out

The family caregivers installed smart door locks on the doors, reversed the door locks, and controlled elevator access, making it difficult for persons with dementia to go out.

“It is to lock the door, hide the key, and prevent him from going out, but he cannot open the door without the key, and keep him in the house.” (P4).

#### Contact neighbors and friends to prevent persons with dementia from getting lost

The family caregivers had previously informed neighbors, friends, and guards that persons with dementia had lost their problems. If a person was found lost, helped take him home.

“Took a photo of him, put the address on the community guard, and asked the community guard not to let him go out of the community.” (P8).

### Theme 3. Using strategies to find lost persons with dementia

After the persons with dementia were missing, the family caregivers used strategies to identify them. This theme contains four subthemes.

#### Let persons with dementia carry contact information

Persons with dementia carry information that could contact their families, such as name tags, personal information sewn on clothes, paper pockets, and information bracelets. Provided information so that others could contact family caregivers when persons with dementia went missing.

“I sewed his name and phone number on every piece of his clothes, whether it was a coat or a shirt, it was sewed on the chest side so that everyone could see, everyone knew that he was an old man with dementia, and he may contact us.” (P20).

#### Electronic device tracking persons with dementia

Mobile phones, GPS watches, and air tags were used to track the location of persons with dementia, allowing family caregivers to track a person’s location at any time and persons with dementia to go out on their own.

“I thought the GPS watch was very useful because you could know where he was. When I watched my dad go out, I determined why my dad had not returned for so long. Just looked at it and knew where he was.” (P20).

#### Find lost persons with dementia in familiar places

If the persons with dementia were lost and did not return home, the family caregivers first visited where the person was accustomed.

“My mother’s behavior pattern and the way she went out are quite fixed…so even if my mother truly disappears, I knew where to find her.” (P15).

#### Call the police to find lost persons with dementia

The family caregivers could not find persons with dementia who were lost or did not return home. Calling the police and seeking assistance.

“Once, when my mother got lost and could not find her, my father went to the police office to call the police.” (P6).

### Theme 4. Exhaustion in long-term care for persons with dementia

The family caregivers did not have an adequate support system and were reluctant to send persons with dementia to long-term care institutions, which allowed them to bear the burden of care for a long time. This theme consists of four subthemes.

#### Long-term stress in supervise persons with dementia

Family caregivers supervise persons with dementia at any time to avoid their loss, which can cause long-term mental stress.

“I had to be with him all day, and pressure was on me. Sometimes, he wakes up at night and opens the door. I knew as soon as he moved, and I had to follow him quickly. I experienced a slight headache due to the pressure. This was too large to sleep well. ” (P16).

#### Insufficient care support from families

Unequal care responsibilities and a lack of replacements in the family system led to family caregivers bearing the burden of care alone.

“I did not think I was filial; I was helpless. My sister married and lived across the street and did not go home to help, and my brother sometimes came back, but he left after eating because I was not married and lived at home. Thus, I became a person who provided care services.” (P10).

#### Conflict between work and care responsibilities

When the family caregivers were at work, they had to deal with the problem of persons with dementia loss. They often had to ask for leave, affecting their work, and they even wanted to retire early to care for persons with dementia.

“My mom got lost, and the police called me; I stopped work immediately to deal with her getting lost. My only pressure was that frequent leave might affect my work.” (P15).

#### Reluctance to send persons with dementia to long-term care institutions

However, the family caregivers worked hard to care for the persons with dementia, they still believe that they have taken better care of themselves. Unable to tolerate poor conditions in which persons with dementia are sent to the institution.

“If she is sent to an institution, some institution personnel may not know her habits. Therefore, I will take care of her by myself, and I will be more at ease in taking care of her by myself.” (P5).

### Theme 5. Coping with the care load

The family caregivers need to care for persons with dementia who have lost their experience. First, they must learn care skills. Caring for persons with dementia is challenging. Using an internal family support system and external care is necessary instead of workers. Let family caregivers rest to reduce their care burden. This theme consists of three subthemes.

#### Learn strategies to prevent persons with dementia from getting lost

Find information on how to deal with losing persons with dementia, such as using an information bracelet, fingerprinting, or GPS.

“Someone told me you should go to the county government to apply for an information bracelet. Later, I learned that there were numbers on the information bracelet. When people found this number and called, they contacted us.” (P14).

#### Family-assisted care work

Children can divide, cooperate, and share their care responsibilities. Provide substantial care and psychological support for persons with dementia and reduce the care burden of family caregivers.

“My brother lived close to his mother and could be responsible for daily life. My job was more flexible, and I was responsible for returning my mother to the clinic. My sister was married; therefore, she could call home to chat.” (P6).

#### Using care replacement workers

Family caregivers use government long-term care resources and foreign workers to give them respite.

“My son had applied for long-term care services, Monday through Friday. Nurse aides went out with him for 2 h every day, and I used this time to go shopping and did other things.” (P16).

## Discussion

Caring for persons with dementia with loss was stressful for the family caregivers. In this study, family caregivers experienced tension, helplessness, and worry when persons with dementia were unexpectedly lost outside the home. Family caregivers accompanied the persons with dementia to prevent them from becoming lost again. The family caregivers developed multidimensional coping strategies to prevent and identify lost persons with dementia. Additionally, the family caregivers learned strategies to prevent persons with dementia from becoming lost, received support from internal family members, and received assistance from external care resources. This helped prevent persons with dementia from getting lost again and improving the care burden of family caregivers. The above findings support the view of Agrawal et al. [14] that getting lost in dementia was a challenge that made care more difficult and that family caregivers must adopt individualized prevention strategies to reduce the risk of getting lost, according to the severity of the dementia, the attitude of the caregiver, and the consequences of getting lost.

The following discussion is based on the study findings.

### Surprised persons with dementia lost outside

Persons with dementia suddenly lost direction in familiar places, or when someone accompanied them, they suddenly disappeared within a short time when they were dealing with other things, and the persons with dementia could not find their way back by themselves. Consequently, familiar places were not as safe as most family caregivers believed. Previous studies also pointed out that neurocognitive deficits often lead persons with dementia to become lost or disappear from safe environments to unknown locations [7]. However, the family caregivers were unaware of the risk of missing persons with dementia until a serious missing event occurred [16]. This study found that, in the process of looking for a person, family caregivers had much anxiety and worry about accidents. Hong and Pai [13] also found similar findings in their research on the family caregivers in dementia. After being lost for the first time, the most noticeable reactions to family caregivers were emotional stress and surprise. Family caregivers were concerned about the safety of the person getting lost (e.g., traffic accidents and personal safety issues [25], and family caregivers expressly may worry that persons with dementia could not be found [26].

### Using strategies to prevent persons with dementia from getting lost

This study found that family caregivers with dementia had someone to accompany persons with dementia at any time when they experienced the persons with dementia ' loss and missing incidents and had fear and worry about being separated from their family members. This was the first strategy adopted by families with dementia for the loss of persons with dementia. Access control should be established to restrict them from going out, and neighbors should be notified to pay attention to restrict to prevent them from becoming lost again. Neubauer and Liu [27] also found that after a severe loss event, family caregivers ' risk perception of loss changes from perceived low risk to high risk. Uses door locks or other methods to prevent persons with dementia from leaving their homes. This results in persons with dementia with limited independence. Additionally, the supervision method of paying close attention to accompanying persons at home has been adopted to manage the safety of persons with dementia and prevent accidents [28]. This may explain why most persons with dementia are lost only once or twice [29].

### Using strategies to find lost persons with dementia

The lost persons with dementia put too much pressure on the family caregivers. However, they still tried to use strategies to find the lost persons with dementia, such as using locator tracking with an active search function or contact information with a passive assistance function and going to the person’s usual place; if they still could not find the persons with dementia, they would call the police to find them. Tu and Pai [5] encouraged persons with dementia to carry contact information even in the early stages of dementia because loss may occur unexpectedly. In addition, in the study of getting lost in dementia, the use of GPS to track persons with dementia often occurs after persons with dementia have been missing for a long time and are found by family members or the police [25]. This study found that GPS technology allowed family caregivers to supervise persons with dementia, reassure them, and enable them to walk safely and independently. Research has also confirmed that safety is more important than protecting autonomy and privacy [30].

### Exhaustion in long-term care for persons with dementia

When persons with dementia were lost, the family caregivers in this study prioritized accompanying and monitoring persons with dementia at any time. They believed that supervising persons with dementia was the most basic strategy to prevent people from getting lost. The family caregivers had been using supervision for a long time, felt too much pressure because of insufficient family support and work, and were reluctant to send persons with dementia to their institutions. This finding supports Chung and Lai’s [31] finding that most family caregivers believed it was essential to supervise persons with dementia. Nonetheless, it was unpredictable for persons with dementia to become lost, staying alert was not feasible, and it was often difficult to stay alert at night, leading to physical and mental exhaustion [7]. When family caregivers with dementia had jobs, their working hours were longer, the workplace lacked flexibility, and it was difficult to coordinate work and care, which affected the quality of life of family caregivers [32]. The burden of family care was heavy, and most family caregivers believed that “being cared for by family members or others” was the best way to care for persons with dementia, mainly because Chinese culture respects unlimited self-sacrifice. “Filial piety” is the core value of traditional Chinese culture and might also be related to some individuals’ fear of being criticized for non-filial piety by their parents [33]. The research results of Connors et al. [34] showed that family caregivers without support or assistance had a higher care load. Over time, family caregivers become additional people and suffer as much as persons with dementia [35].

### Coping with the care load

Caring for lost persons with dementia brings trouble to the family. Nevertheless, obtaining informational support for coping with getting lost, internal family support, external care instead of workers, and substantive support would improve the care load. These results support Xu et al.’s [36] point of view that social support has a significant direct relationship with the burden of family caregivers. Regarding informational support, dementia education interventions could help family caregivers prepare to care for persons with dementia by understanding dementia symptoms and how to deal with them [37]. Tu and Pai [5] noted that the loss of persons with dementia was unpredictable and occurred rapidly. It is essential to teach caregivers to take preventive measures early, and it is recommended that they carry locators as soon as possible. Furthermore, family caregivers must provide substantive support to obtain information. Family is the most crucial source of support for family caregivers. Kimura et al. [17] pointed out that family members helped each other, and emotional support and substantive support were factors that could reduce the burden on caregivers. However, it has become increasingly difficult for family members to provide care due to social changes such as a declining birth rate, longer life expectancy, and changes in family structure. Therefore, this study found that using the long-term care resources provided by the government and hiring foreign care workers are essential Shih et al. [38] spousal caregivers have less access to long-term care-related information and utilize services less frequently compared to child caregivers. Therefore, information about long-term care services is the most important factor. Studies have shown that the longer the dementia and worse the cognition of persons with dementia, the greater the use of foreign care workers [38]. In a study by Peng et al. [39], foreign care workers improved the mental fatigue of family caregivers when persons with dementia were lost outside the home. Moreover, family caregivers can receive substantial help from family members and other social groups, which could reduce the burden of caring for persons with dementia and shared care tasks, allowing family caregivers to obtain respite services [35].

### Limitations

The researchers attempted to obtain data on ages, sexes, and relationships with persons with dementia. However, this qualitative study involved 20 participants from northern Taiwan. Due to a lack of resources, researchers could not conduct research across various hospitals, day care centers, and countries. Additionally, this study did not separately discuss the care experiences of persons with different dementia levels and times of loss. In the future, the care experiences of family caregivers with varying levels of dementia and lost time can be presented. Finally, future research should focus on family caregivers and persons with dementia, comparing their experiences of getting lost, caring for them, and the use of loss prevention strategies.

## Conclusions

This study showed that losing persons with dementia caused a heavy psychological burden on family caregivers. They also realized that the person could not go out safely and adopted strategies to prevent persons with dementia from being lost. The first strategy was to accompany the person at any time, which also caused family caregivers to be overloaded with stress. This study also found that family caregivers needed to learn strategies to prevent persons with dementia from becoming lost and provide resource support to improve caregivers’ load. The results of this study could help medical staff understand the experiences of family caregivers in caring for lost and demented family members, formulate training plans, and teach family caregivers to adopt appropriate strategies to prevent persons with dementia from becoming lost. Continuous provision of long-term care services increases the burden on family caregivers in dementia care.

### Relevance to clinical practice

In the future, teaching caring skills for family caregivers with dementia should focus on family caregivers, realizing that persons with dementia may be lost in unexpected situations. They need to be prepared in advance to learn strategies to prevent loss. Furthermore, they should choose suitable strategies used by family caregivers and families to reduce the risk of persons going out alone and getting lost. They must help lost individuals find their way home as soon as possible. Additionally, care is a long and arduous process with great physical and mental pressure. The results show that government long-term care resources and foreign care workers reduce the burden on family caregivers. Therefore, Taiwan’s Ministry of Health and Welfare provides various long-term care resources to help family caregivers reduce their burden of care. However, it is necessary to use multiple channels (e.g., the internet, television, and newspapers) to publicize long-term care resources, increase family caregivers’ awareness of long-term care policies, and use long-term care services to improve the burden on family caregivers. Finally, do the family members provide care and support? This was an essential factor for the family caregivers to feel exhausted and to cope with the burden of care. Therefore, dementia management specialists should assist family caregivers in organizing family care resources and helping them use family and government long-term care resources to improve the quality of care.

## Data Availability

The data data cannot be shared openly, for example to protect study participant privacy and are available from the corresponding author upon reasonable request.
